# Generation and evaluation of IgY-scFv based mimetics against canine parvovirus

**DOI:** 10.1186/s13567-021-00943-9

**Published:** 2021-05-13

**Authors:** Shikun Ge, Xingxing Zhang, Fagang Zhong, Xiang Liu, Xiaoying Zhang

**Affiliations:** 1 Chinese-German Joint Laboratory for Natural Product Research, Key Laboratory of Biological Resources and Ecological Environment of Qinba Areas, School of Biological Science and Engineering, Shaanxi University of Technology, Hanzhong, China; 2 Centre of Molecular and Environmental Biology, University of Minho, Campus de Gualtar, 4710-057 Braga, Portugal; 3 Department of Biomedical Sciences, Ontario Veterinary College, University of Guelph, Guelph, ON Canada; 4 State Key Laboratory for Sheep Genetic Improvement and Healthy Production, Xinjiang Academy of Agricultural and Reclamation Science, Shihezi, China

**Keywords:** Antibody mimetics, IgY-peptide, Canine parvovirus (CPV), IgY-scFv, IgY technology

## Abstract

Antibody mimetics may be used for various biomedical applications, especially those for which conventional antibodies are ineffective. In this study, we developed a smaller molecular chicken IgY mimetic peptide (IgY-peptide) based on the complementarity-determining regions (CDRs) of the anti-canine parvovirus (CPV) IgY-scFv prepared previously. The mimetic peptide showed no cross-reactivity with canine distemper virus (CDV) and canine coronavirus (CCV) and showed excellent protective properties for Crandell-Rees Feline Kidney (CRFK) cells against CPV. This study is the first attempt to develop a mimetic IgY-peptide and demonstrates the ease and feasibility in generating such a novel antibody-like functional molecule for biomedical purposes.

## Introduction, methods and results

Numerous attempts have been successfully made to generate avian sourced monoclonal IgY (mIgY) or recombinant functional IgY fragments, particularly the single chain fragment variables (scFv) [1, 2], for immunological detection [3], diagnostic and therapeutic purposes [4] in veterinary and human medicine.

In our recent study [4], hens were immunized with virus-like particles (VLP) of canine parvovirus VP2 (CPV-VP2) and the specific IgY-scFv were generated by using the T7 phage display system. This result confirmed the feasibility of generating diversified avian IgY-scFv libraries against pathogenic targets of interest for both diagnostic and therapeutic purposes. However, scFv is still relatively large in molecular weight, complex in the states of spatial structure and charge, and easy to form aggregations, however, such limits could be overcome by designing antibody like small molecules, the antibody mimetic peptide, which retains the characteristics of the specific antibody to the maximal extent, while referenced the advantages and convenience of peptides [5].

After polyclonal antibodies, full-length monoclonal antibodies and recombinant antibody fragments, antibody-based mimetics (peptides) have been considered as the fourth generation of antibody engineering, enabling the optimized and easy design of target molecules of interest, easy production of large amount of mimetics based on peptide design and synthesis, higher molecular stability for better biomedical applications, and an alternative to animal-based antibody generation strategies [5]. The current study aimed to generate anti-CPV VP2 IgY-scFv based mimetics and to evaluate the feasibility of such mimetics as a specific antibody-like functional molecule.

**Figure 1:**
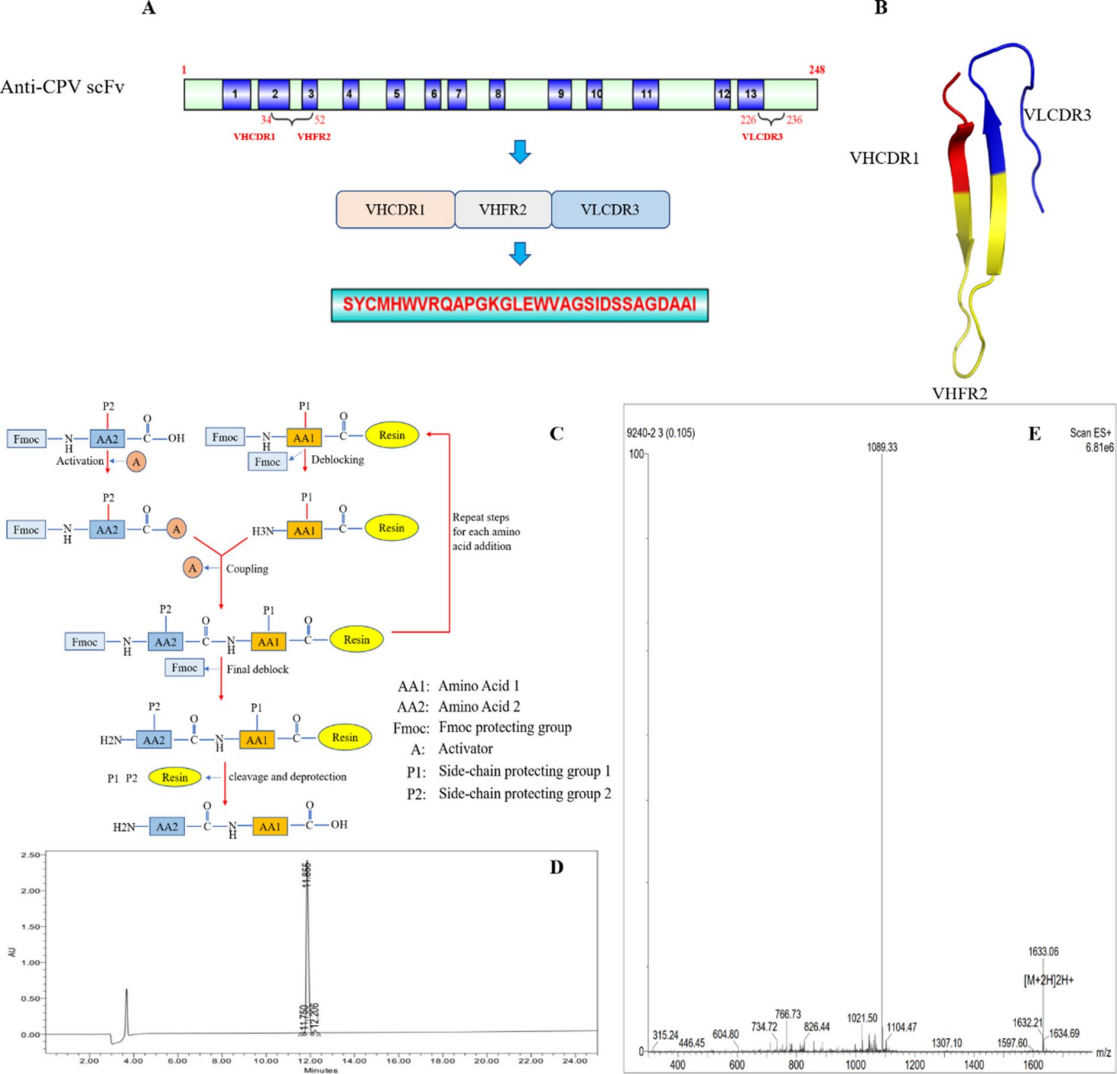
**The design and synthesis of IgY-peptide. A** Obtained anti-CPV IgY-scFv screened by T7 phage display technology which had confirmed specificity and neutralization ability; A total of 13 epitopes were predicated on scFv sequence by PREDICTED ANTIGENIC PEPTIDES software (UCM, Madrid, Spain). **B** The three-dimensional protein structure model was obtained by I-TASSER software (a hierarchical approach to protein structure and function prediction; UMich, Ann Arbor, MI, USA). **C** Solid phase peptide synthesis scheme. IgY-peptide was synthetized using solid phase Fmoc on a PEG-Polystyrene support resin. Upon synthesis completion, side chain protecting groups were removed and the peptide was simultaneously cleaved from the resin. The cleaved and deprotected peptide material was further precipitated, washed and dissolved in a buffer containing H_2_O/ACN/HOAC prior to lyophilization. **D** The determination of IgY-peptide by HPLC. Retention time of mimetic peptide: 11.855 min; Analytical column type: SHIMADZU Inertsil ODS-SP (4.6 × 250 mm × 5 µm); Pump A: 0.1% trifluoroacetic in 100% water, Pump B: 0.1% trifluoroacetic in 100% acetonitrile; Wavelength: 220 nm. **E** MS analysis of purified mimetic peptide. ESI_MS. value = (M + n*H)n, M is the correct mass, n is an integer and *n* > 0, while n is range.

This study builds on our findings previously published [4], CDR and framework region sequences of our previously obtained IgY-scFv against the VLP of CPV-VP2 (Figure 1A) [4] were used to create an antibody mimetic peptide comprising two interacting V_H−_ (VHCDR1) and V_L−_ (VLCDR3) derived CDRs (31 AA, VHCDR1-VHFR2-VLCDR3). The framework region (VHFR2) from either the V_H_ or V_L_ domain orients the two CDRs in a manner resembling their disposition in native molecules (Figures 1A and B). As the CDR3 loops of the V_L_ domains are the accessible entities that interact with antigenic epitopes, we defined the CDR3 domain of the V_L_ domain of a Fab fragment as the key component of our prototype antibody mimetic, the CDR1 loop of the V_H_ domain was selected as the complementary component. The IgY-scFv mimetic peptide (IgY-peptide) was synthesized using a solid phase method (Figure 1C). The purity of IgY-peptide was 98.30%, which was determined by HPLC (Figure 1D), the molecular weight of IgY-peptide was analyzed by mass spectrometry (MS) (Jietide Biotech, Nanjing, China) (Figure 1E).

**Figure 2:**
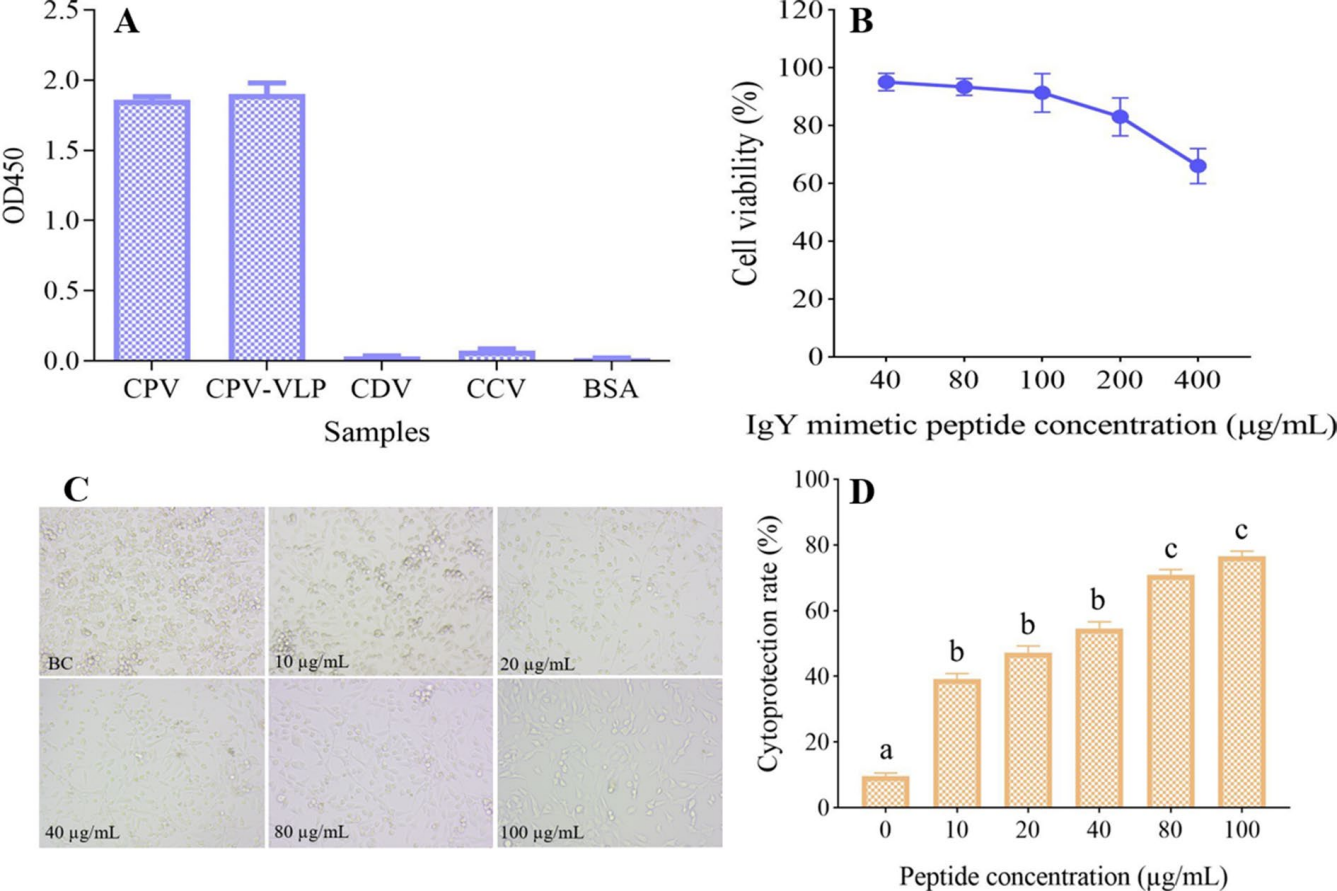
**The protective rate of IgY-peptide against CPV-infected cells. A** The specificity analysis of mimetic peptide. Control group: bovine serum albumin (BSA). **B** Cytotoxicity of increasing IgY-peptide concentrations on CRFK cells. **C** Healthy cells (100 µg/mL): shuttle shape; cytopathic effect (CPE; BC): cells rounded, detached from bottom and lysed (400×). BC, blank control, the peptide replaced by PBS. **D** Results are means of three replicates (Mean ± SD), different letters represent significant differences (*p* < 0.5).

To evaluate the specificity of IgY-peptide in the detection of CPV, a total of 28 clinical dog stool samples were collected using sterile swabs in an animal hospital (Xinger, Xi’an, China). Among them, there were 24 positive samples and four negative samples (samples number 4, 6, 12 and 26) confirmed by a commercial colloidal gold test strip (ICA). Each sample was homogenized (10%, w/v) in PBS (1 mL, pH 7.2) and centrifuged, then the supernatants were analyzed by ELISA and PCR [4]. There was no difference in the detection performance of IgY-peptide and IgY-scFv on CPV clinical fecal samples. The IgY-peptide detection results were positive in some of the samples (samples number 5, 11, 15, 20, 21 and 28), and its detection value was not as high as IgY-scFv (Table 1). The IgY-peptide had no cross-reactivity with CDV and CCV (Figure 2A).

**Table 1:** **Comparison of IgY-scFv and IgY-peptide mimetic detection on clinical samples**.

Sample	IgY-scFv (Mean ± SD)	IgY-peptide (Mean ± SD)	ICA	PCR	Sample	IgY-scFv (Mean ± SD)	IgY-peptide (Mean ± SD)	ICA	PCR
1	0.52 ± 0.02	0.32 ± 0.00	+	+	16	0.51 ± 0.01	0.48 ± 0.12	+	+
2	0.52 ± 0.01	0.46 ± 0.03	+	+	17	0.85 ± 0.03	0.99 ± 0.17	+	+
3	0.59 ± 0.01	0.52 ± 0.02	+	+	18	0.67 ± 0.01	0.59 ± 0.13	+	+
4	0.14 ± 0.03	0.05 ± 0.00	−	−	19	0.71 ± 0.01	0.54 ± 0.16	+	+
5	1.51 ± 0.00	0.35 ± 0.04	+	+	20	0.84 ± 0.05	0.31 ± 0.05	+	+
6	0.11 ± 0.02	0.03 ± 0.01	−	−	21	0.67 ± 0.01	0.32 ± 0.04	+	+
7	1.99 ± 0.06	1.56 ± 0.35	+	+	22	2.2 ± 0.13	1.43 ± 0.27	+	+
8	0.56 ± 0.03	0.64 ± 0.02	+	+	23	0.87 ± 0.01	0.59 ± 0.18	+	+
9	0.82 ± 0.00	0.66 ± 0.03	+	+	24	0.45 ± 0.01	0.32 ± 0.02	+	+
10	1.1 ± 0.05	0.96 ± 0.03	+	+	25	0.58 ± 0.00	0.93 ± 0.03	+	+
11	1.47 ± 0.08	0.74 ± 0.04	+	+	26	0.15 ± 0.02	0.04 ± 0.00	−	−
12	0.14 ± 0.03	0.06 ± 0.01	−	−	27	0.33 ± 0.00	0.51 ± 0.19	+	+
13	0.83 ± 0.03	0.77 ± 0.03	+	+	28	1.98 ± 0.01	0.96 ± 0.03	+	+
14	0.86 ± 0.01	0.57 ± 0.05	+	+	NC	0.03 ± 0.02	0.07 ± 0.01		
15	0.84 ± 0.04	0.22 ± 0.04	+	+					

“+” the ICA positive samples, “−” the ICA negative samples; NC, negative control, the sample was replaced by BSA. Statistical principle, P/N > 2.0 (P samples value; N NC value), it can be judged as positive at the level of 99.9%. Results are means of three replicates. The PCR and ICA results were in accordance to [4].

Crandell-Rees Feline Kidney (CRFK) cells were trypsinized and plated in a 96-well plate at a density of 5 × 10^3^ cells/well. The cells were grown in Dulbecco’s Modified Eagle Medium (DMEM) containing 10% Fetal Bovine Serum (FBS) overnight at 37 °C in 5% CO_2_ incubator. The cells were treated with increasing mimetic peptide concentrations followed by a cell proliferation assay. The CCK-8 assay kit (Beyotime, Shanghai, China) was used to measure the cell viability in different concentrations of the mimetic peptide. Cells in medium without the IgY-peptide were considered as positive control for the viability assay and monitored at 24 h. The CCK-8 cell proliferation assay protocol was followed, and the absorbance was recorded at 450 nm using a microplate reader (Beyotime, Shanghai, China). The IgY-peptide had no toxicity to cells (cell survival rate >90%) (Figure 2B), when its concentrations were below 100 µg/mL. We can therefore determine that a concentration (≤100 µg/mL) is suitable for subsequent cellular studies.

The ability of the IgY-peptide to inhibit virus growth on the CRFK cells was determined. CRFK cells were grown in six-well microplates (1.5 × 10^6^ cells/well) for 24 h. Cell culture media were removed, and the cells were washed three times with PBS. Then, new media containing virus supernatant mixed with increasing concentrations (10, 20, 40, 80 and 100 µg/mL) of IgY-peptide were separately added into the cells and incubated for 24 h at 37 °C. The CCK-8 cell proliferation assay protocol was followed, and the absorbance was recorded at 450 nm using a microplate reader (Beyotime, Shanghai, China). Cells (BC) became significantly rounded, broke away from the bottom, and broke apart into fragments. With the increasing mimetic peptide concentration, the cells state was significantly improved, and most of the cells showed shuttle shape when the concentration of IgY-peptide was 100 µg/mL (Figure 2C). The cell protection rate showed a IgY-peptide dose-dependent type (Figure 2D).

All experiments were performed with at least three independent experiments. Statistical significance was determined by the Student *t* test when two groups were compared, or by one-way analysis of variance (ANOVA), when more than two groups were compared. *p* < 0.05 was considered to be statistically significant.

## Discussion

Antibodies exhibit versatility and have a broad spectrum of use as powerful tools in research, detection, diagnosis and therapy as they, in theory, can direct to any desired target. Antiserum (IgY from avian egg yolk) based polyclonal antibody, hybridoma technique based full-length monoclonal antibody and genetically engineered antibody fragments and chimeras can be considered as three generations of antibody engineering which exhibit different characteristic, advantages and limits (Table 2), as well as suitable application areas. Antibody mimetics is a promising alternative to animal sourced antibodies which has developed in the last decade arising from advances in protein and peptide design and synthesis, and better understanding of antibody function and structure [5].

**Table 2:** **Antibody generation platforms and their feature**.

Antibody type	Stability	Specificity	Homogeneity	Reproducibility	Representative technology
Polyclonal antibody	+++	+	+	+	Mammal antiserum, avian IgY technology
Monoclonal antibody	++	+++	++	++	Hybridoma technique
Recombinant antibody (fragment)	+	+++	+++	+++	Phage display
Antibody mimetics	+++	++	+++	+++	Protein direct evolution, CDR-FR fusion

The generation of avian sourced monoclonal IgY (mIgY), particularly IgY-scFv, combines the advantages of avian IgY antibodies and features of monoclonal antibodies (mAbs), with validated potential for immunological detection and diagnosis, for screening and validating biomarkers and for therapeutic uses in both veterinary and human medicine [1, 2].

In the current study, we designed the antibody mimetic based on three major antigen-binding entities (VHCDR1, VHFR2 and VLCDR3) that reside in a variable domain of either the heavy chain (V_H_) or light chain (V_L_) of the antigen-binding fragment (Fab) of antibody (Figure 1) [5, 6]; despite it is accepted that antigen recognition by whole antibodies requires multiple noncovalent forces involving all six CDR loops residing in the V_H_ and V_L_ domains, and the contributions of synergic interactions of V_H_ and V_L_ domains are not well understood [6]. To simplify the peptide design, we designed the IgY-peptide in reference to previous study [6], However, it could be of interest to construct phage display peptide libraries and applied to the epitopes analysis of a specific monoclonal antibody with affinity selection of a peptide-mimetic, to optimize the peptide by screening and selecting antigen-recognition sites in more complete domains based on the entire IgY-scFv sequence. The efficacy of such selected peptide could be further increased and the structure could be optimized, which should be a research direction in the future [7]. Instead we chose a relatively simpler mimetic design by using the following principles: firstly, as antigen recognition by intact Fab is synergistically produced by all six CDRs residing in both the V_H_ and V_L_ domains, it should contain at least two antigen-binding sites: one from the V_H_ and the other from the V_L_ domain. Such synergistic recognition cannot be accomplished if the CDR loops all originate from one variable domain [6, 8]. Secondly, as the CDR3 loop is the central of Fab, most accessible antigen binding segment in an intact Fab, it should be regarded as an essential component of the mimetic [9–11]. Thirdly, the CDR3 loop should be complemented by either the CDR1 or CDR2 loop of the other variable domains, as these are normally the closest to CDR3 in the parent antibody. Fourthly, the C-terminus of the selected CDR1 or CDR2 loop and the *N*-terminus of the selected CDR3 loop should be joined with a framework region selected from either the V_H_ or the V_L_ to approximate the linkage of CDR in the parent molecule.

Apart from general advantages of antibody mimetics [5], IgY-peptides may offer additional superiority as specific antibodies against mammalian conserved targets can be better obtained by using IgY technology [1]. A deeper and better understanding of IgY molecule, IgY affinity maturation and IgY glycosylation may be helpful to provide further insights into rational IgY-peptide design.

As a pilot study, we conclude that avian IgY-scFv based mimetics can be designed and generated with confirmed specificity and neutralization ability, which enables diversified antibody engineering and further development of IgY technology.

## Abbreviations

CPV: Canine parvovirus
VLP: Virus-like particles
scFv: Single chain fragment variables
ICA: Immunochromatographic assay
CDV: Canine distemper virus
CCV: Coronavirus
mAbs: Monoclonal antibody
IgY-scFv: Chicken IgY single chain variable fragments
CRFK: Crandell-Rees feline kidney
DMEM: Dulbecco’s modified eagle medium
CDRs: Complementarity-determining regions
BSA: Bovine serum albumin
FBS: Fetal bovine serum
mIgY: Monoclonal IgY
MS: Mass spectrometry
HPLC: High performance liquid chromatography
